# Higher Prevalence of Human Papillomavirus Infection in Adolescent and Young Adult Girls Belonging to Different Indian Tribes with Varied Socio-Sexual Lifestyle

**DOI:** 10.1371/journal.pone.0125693

**Published:** 2015-05-08

**Authors:** Kirti Sharma, Atul Kathait, Asha Jain, Karmila Kujur, Shirish Raghuwanshi, Alok Chandra Bharti, Asha Chandola Saklani, Bhudev Chandra Das

**Affiliations:** 1 Dr. B. R. Ambedkar Centre for Biomedical Research, University of Delhi, Delhi, India; 2 School of Biosciences and Clinical Research, ApeejayStya University, Sohna-Palwal road, Sohna, Gurgaon, India; 3 Sri MaaSardaArogyaDham, Raipur, Chhattisgarh, India; 4 Department of Obstetrics & Gynaecology, M.G.M. Medical College & Hospital, Sakchi, Jamshedpur, Jharkhand, India; 5 Department of Obstetrics & Gynaecology, District Hospital, Harda, Hoshangabad, Madhya Pradesh, India; 6 Division of Molecular Oncology, Institute of Cytology & Preventive Oncology (ICMR), Noida, India; Banaras Hindu University, INDIA

## Abstract

**Background:**

Despite high prevalence of human papillomavirus (HPV) infection and cervical cancer in Indian women, no study has been done in tribal populations whose socio-sexual lifestyle is different. Therefore, HPV screening has been carried out in pre-adolescent, adolescent and young adult tribal girls using self-collected urine samples.

**Methods:**

20–35 ml self-collected midstream urine samples were obtained from a total of 2278 healthy tribal girls (9–25 years) comprising pre-adolescent, adolescent and young adults from three Indian states: Madhya Pradesh, Jharkhand and Chhattisgarh. β-globin positive 2034 samples were employed for HPV detection and genotyping.

**Results:**

The overall prevalence of HPV infection in tribal girls was 12.9% (262/2034). More than 65% (172/262) of them were infected with HR-HPV types of which HPV16 was the most predominant type (54%). Young adult girls aged 18–25 years showed a significantly higher prevalence of HPV infection (19.2%; OR = 3.36; 95% CI 2.97–6.34, P<0.001) as compared to that in adolescent (11.4%; OR = 1.82; 95% CI 1.20–2.76, P<0.01) or pre-adolescent girls (6.6%).

**Conclusion:**

This is a first study showing significantly a very high prevalence of HPV infection in adolescent and young adult tribal girls possibly due to different socio-sexual behavior, indicating a serious health concern for Indian tribal women.

## Introduction

Infection with specific types of high risk human papillomaviruses (HR-HPVs) is proved to be the principal etiologic agent for the development of cervical cancer which is the most predominant cancer in Indian women. Several well known risk factors such as early age of sexual intercourse, poor genital hygiene, multiple sexual partners, multiple pregnancies, high parity are associated with the development of cervical cancer [1–3]. More than 80% of Indian women with cervical cancer harbor HR-HPV types 16 and 18 [4–7]. Following establishment of causal link between HPV infection and cervical cancer, two vaccines Gardasil (MSD Merck & Co., Inc. Whitehouse station NJ, USA) and Cervarix (GlaxoSmithKline Biologicals, Belgium) have been developed against the two most common cancer causing genotypes HPV16 and HPV18 and are highly effective in preventing above HPV infection [8]. The vaccines are preferably given to adolescent girls or females aged between 9–26 years [9] before acquiring HPV infection.

Adolescent girls are unique and vital group for investigating socio-demographic and sexual factors responsible for acquisition of HPV infection as their cervix is immature having larger area of ectopy and sexual exposure predisposes them to various reproductive tract infections including HPV infection. However, HPV infection has also been detected in sexually inactive pre-pubertal girls, which may be due to non-penetrative sexual behaviors [10,11], autoinoculation [12], fomites [13], and other non-sexual routes [14]. Previous studies showed that the risk of HPV infection increases soon after the onset of sexual activity [15,16] thus, prevalence of HPV infection has been reported to be highest in young women which later declines during second and third decades of life [17,18]. Yet, most infections are transient and cleared within a few months, but if it persists for a year or two or reappears, the lesions may progress to higher grade dysplasias and invasive cancer.

Of numerous biological samples employed for detection of HPV, urine is particularly attractive as a simple, noninvasive method of screening female adolescents as pelvic examination might not be feasible and ethically acceptable for these age group girls [4, 19–22]. Since there is no data available on HPV prevalence in pre-pubescent and young adult tribal women from different tribal regions of India which is a home for more than a half of the world’s tribes, we have used urine sampling for screening HPV infection in these tribal population.

Tribal people generally follow isolated lifestyle in deep forest and practice their own specific customs and rituals. The sexual activity starts at an early age and polygamy is well tolerated and these lead to multiple partners and early and multiple pregnancies. Delivery of child occurs at home under unhygienic conditions without medical help or nursing. Common factors like malnutrition, poor hygiene, lack of education, low economic status, unawareness and absence of modern medical facility make tribal people most vulnerable to several chronic and infectious diseases such as sexually transmitted diseases and cancer [23, 24].

Although, several HPV studies are available on general population, till date no study has been conducted on tribal population whose lifestyle and sexual behavior are completely different from mainland inhabitants. Therefore, the present study was carried out to examine the status of HPV infection and its genotype distribution in pre-adolescent (9–12 yr), adolescent (13–17 yr) and young adult girls (18–25 yr), from three different tribal states of India.

## Materials and Methods

### Study area and subjects

The study was carried out from March to December 2012 on tribal girls aged between 9 to 25 years belonging to different tribes from three different states of India–Madhya Pradesh (Hoshangabad), Chhattisgarh (Narainpur and Kondagaon) and Jharkhand (Jamshedpur). These tribes were very poor and are mainly dependent on forest produce and primitive agriculture.

### Demographic data and sample collection

A total of 2278 urine samples along with socio-demographic data were collected by home to home visits and from local schools. Subjects were interviewed in private by face to face interview by trained nurse/ social worker at participant’s home or in the school. Interviews were conducted in local language using a pretested questionnaire which includes socioeconomic and demographic details, sexual behavior, genital hygiene, awareness about HPV and cervical cancer. Self-collected 20–35 ml mid-stream urine was collected in 50 ml sterile falcon labeled with unique identification number, name, age, father’s/ mother’s name and place. To obtain sufficient cells, at least 20ml of urine sample was centrifuged at 5000 rpm for 10 min at 4°C and the cell pellet obtained was washed twice with 1x PBS (phosphate buffered saline) and stored at −20°C until analysis. Participants aged between of 9 to 25 years, who understand local (Hindi) language, had given written informed consent, had no psychiatric disturbances, no fever or not undergoing menstruation and had permission from parents for participation were included in the study. Face to face interviews were carried out in a very friendly atmosphere to elicit personal details such as menstrual period, genital hygiene, sexual habits, sexual partners, pregnancy etc. The socio-demographic data were collected with respect to their age, education, income and awareness about HPV and cervical cancer.

### Ethical consideration

The study was approved by the ethics committee of Dr. B. R. Ambedkar Center for Biomedical Research (ACBR), University of Delhi, Delhi in accordance with the Helsinki Declaration. Written informed consent was obtained from all the participants or the next of kin, caretakers or parents/guardians on behalf of all the minors/ children enrolled in our study. Additionally, the study was explained orally in local language by the female health workers and gynecologists to all participants. It was assured that in case of early symptoms of the disease and for follow up the participants should report to the nearest Government hospitals which are collaborating with this study and the treatment will be provided free of cost. Though, strict confidentiality of results was maintained, the participants positive for oncogenic HR-HPV types were informed and were asked to come for follow-up once in a year.

### HPV detection and genotyping

Genomic DNA was extracted from urine samples using standard proteinase K digestion, phenol/chloroform extraction method and ethanol precipitation [4, 25, 21]. β-globin PCR was used as an internal control to confirm the absence of inhibitors, integrity, quality and adequacy of DNA extracted from urine samples. Also, for each PCR experiment, a positive (HPV 16 DNA extracted from SiHa cell line) and a negative control (distilled water) were used. HPV analysis was done by putting two independent PCR using HPV L1 consensus primers MY09/MY11 and GP5+/GP6+ that gave 490bp and 150 bp amplicons respectively. HPV genotyping was done by PCR using type-specific primers for predominant HPV types 16, 18, 6, and 11. In order to detect other high risk and low risk HPV genotypes, reverse line blot (RLB) assay which detects 37 HPV genotypes as recommended by WHO’s HPV Lab Net Program was employed using PGMY09/11 primers according to WHO HPV laboratory manual [26, 27]. For final confirmation the PCR products were directly sequenced on an automated DNA sequencer (310 ABI Prism genetic analyzer; Applied Biosystem, U.S.A) according to the manufacturer’s protocol.

### Statistical analysis

Statistical analysis was performed to estimate the prevalence of HPV infection and its association with various risk factors. According to age tribal girls were divided into three categories: pre-adolescent (9–12 years), adolescent (13–17 years) and young adult girls (18–25 years). Univariate analysis was performed to estimate the unadjusted odds ratio with 95% confidence interval (CI). Binary logistic regression was used to estimate age adjusted odds ratios and corresponding 95% confidence intervals for the association between various factors and HPV infection. Multivariate logistic analysis was used to analyse that among various factors studied which is more closely associated with HPV infection. P value<0.05 was considered statistically significant. The sensitivity, specificity, positive predictive value (PPV) and negative predictive value (NPV) of HPV detection in urine sample were also calculated using standard statistical formula. All analysis was carried out using SPSS (version 18.0 for Windows; SPSS Inc., Chicago, IL).

## Results

### Sociodemographic characteristics and socio-sexual behavior

**Fig 1:**
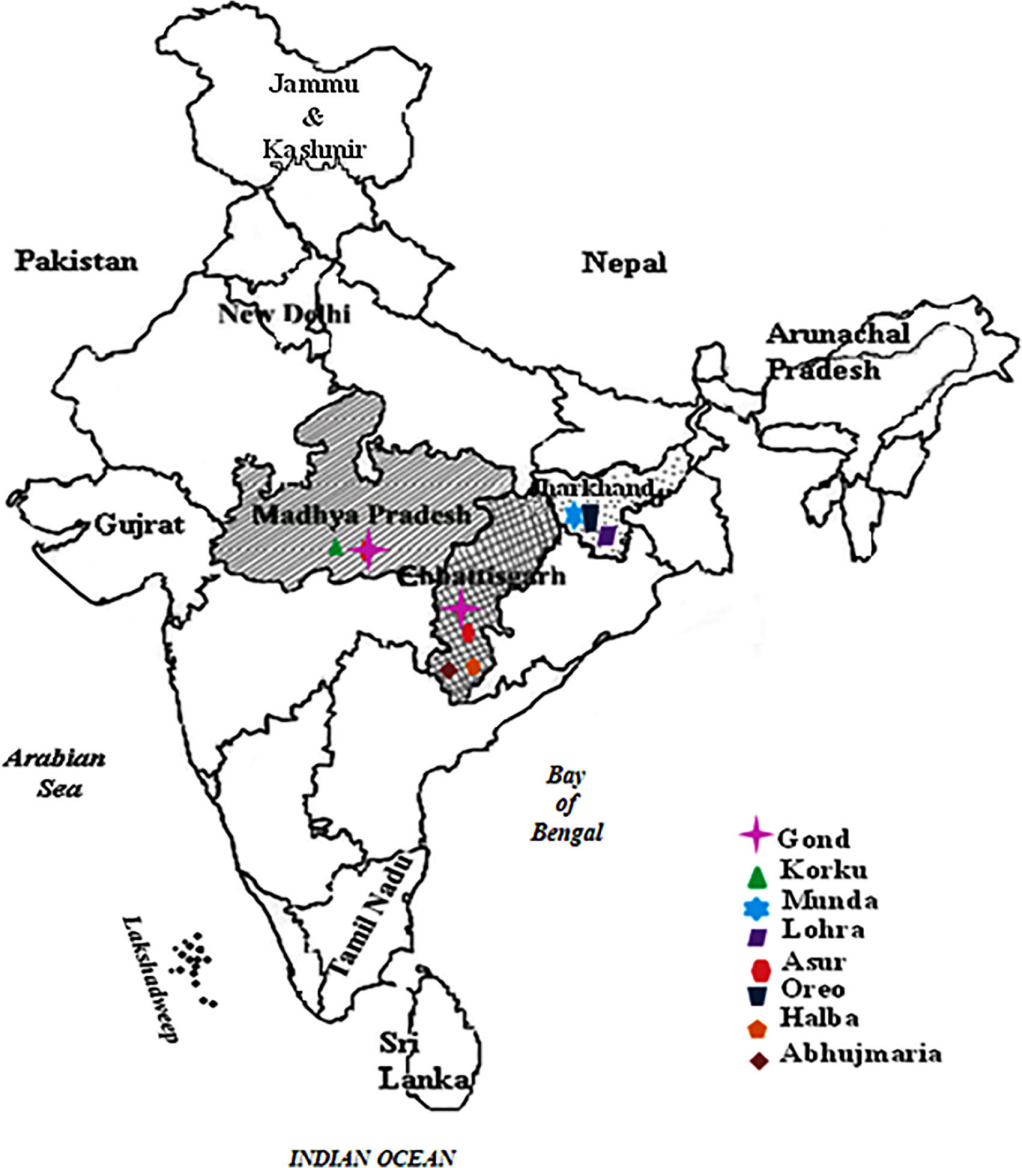
Map of India showing location of three different states (shaded areas) where the study was conducted on eight different tribes. The locations of different tribal communities have also been shown by specific symbols.

In the present study 8 different tribes from three different Indian states namely: Madhya Pradesh, Jharkhand and Chhattisgarh were investigated. The type of tribes were Gond and Korku from Madhya Pradesh and Munda, Lohra, Asur and Oreo from Jharkhand while Muria, Gond, Halba and Abhujmaria from Chhattisgarh (see Fig 1). These tribes were not only geographically distinct but also had their own unique customs, traditions, believe, social and sexual practices. It was observed that health problems like anemia, underweight, malnutrition, pneumonia, tuberculosis and malaria were most common among them. They hardly visit any public health clinic or hospitals as they strongly believe in their Gods and Goddesses for their health and use variety of plants and herbs from the forest for the treatment of their various ailments and diseases. It was observed that tribal girls get involved in sexual activities from an early age and more so after achieving menarche at as early as 10–12 year that leads to early pregnancy, higher parity, abortions and child birth at regular intervals of a year or so. Polygamy and multiplicity of sexual partners were common among them and well tolerated in the community. There is no system of formal marriage but they have freedom of selecting their partners during a special community festival and live together without getting married (23). They generally lack clean drinking water and health care facilities. Their staple food is rice and they consume a lot of locally fermented (alcoholic) beverages’ called mahua and tadi which have strong toxic effects.

### Cohort description

**Fig 2:**
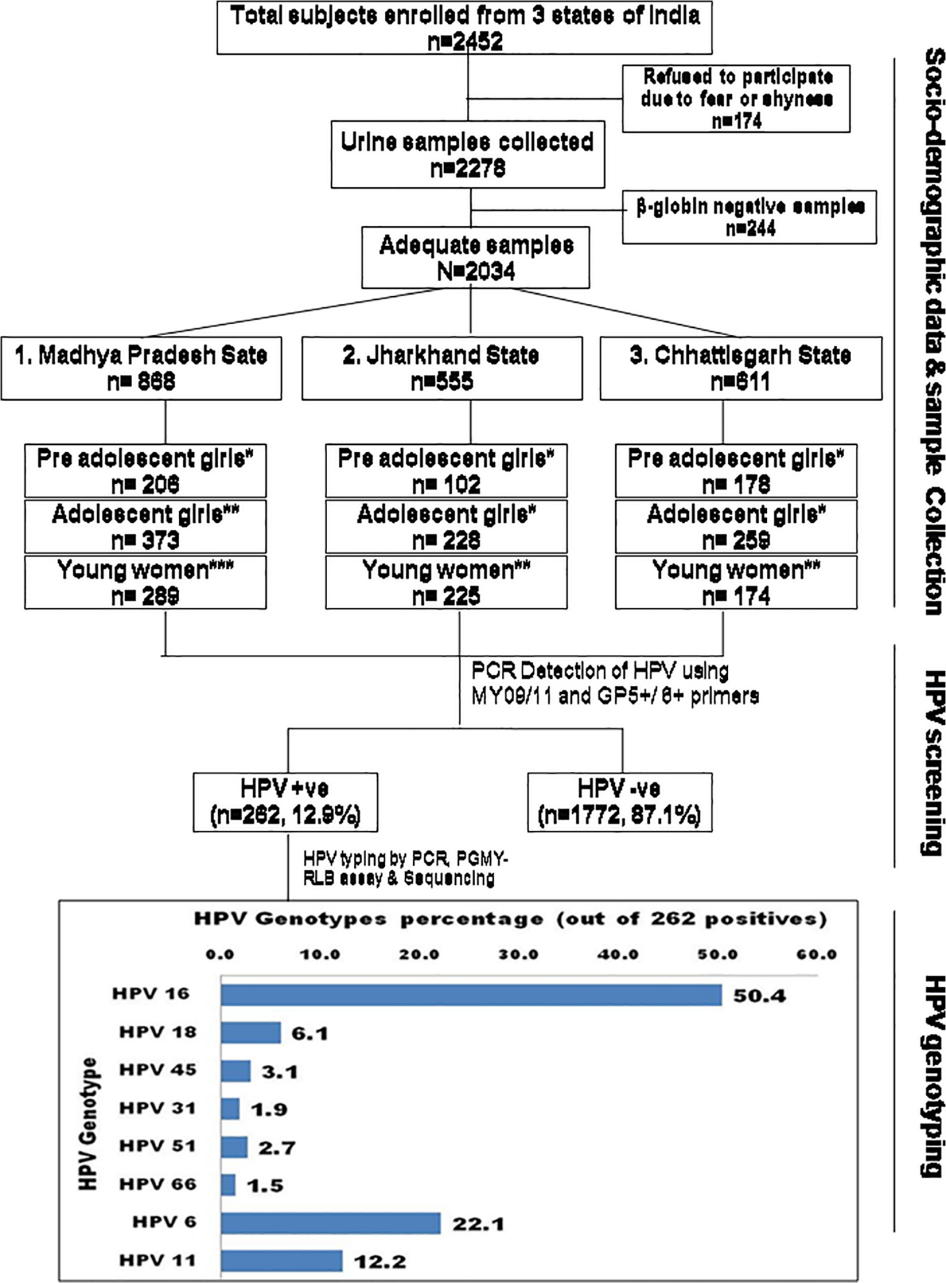
Flow diagram showing design and outcome of the study. * 9–12 years, ** 13–17 years, ***18–25 years.

Initially 2452 tribal girls aged between 9–25 years were approached for the study but 174 of them refused to participate due to some personal reasons such as shyness, lack of parental permission, fear of discovering serious diseases etc. Therefore, a total of only 2278 girls participated and self-collected urine samples were obtained from them. Out of these, 2034 samples which showed adequate DNA and successful amplification of β globin gene were subjected to HPV detection and genotyping (see Fig 2).

Among the tribal girls enrolled in this study 868 (42.7%) lived in Madhya Pradesh, 555 (27.3%) in Jharkhand and 611 (30%) in Chhattisgarh. All the participants were further categorized into (i) pre-adolescent 9 to 13 years (486; 23.9%), (ii) adolescent 13–18 years (860; 42.3%) and (iii) young adults 18–25 years (688; 33.8%) (see Table 1). At the time of study 43.6% pre-adolescent girls, 75.9% adolescents and 100% young adult girls have achieved menarche. Ninety two percent young adult girls, 77.1% adolescent and 36.8% pre-adolescent girls accepted that they have had boyfriends. Hereboyfriend means a male partner with whom the participants have sexual relation (penetrative sex) or non-sexual relationships (touching of private parts etc.) These tribes live isolated in forest and totally depended on forest produce and agriculture for their daily livelihood. A total of 1036 (50.9%) tribal girls’ families were placed under low income group whose monthly income was less than 1000 INR = $20.00 but there was no family having income more than Rs 3000 ($ = 60.00) per month which indicates an extremely poor economic status of tribal population. As regards education, 43.8% of girls were illiterate, but only 9.6% attained the higher education that was upto 12th standard only (see Table 1).

**Table 1:** Prevalence of HPV infection in different tribal populations from three states of India.

Categories	Madhya Pradesh		Jharkhand		Chhattisgarh		Overall	
	No of cases (%)	HPV +ve n (%)	No of cases (%)	HPV +ve n (%)	No of cases (%)	HPV +ve n (%)	No of cases (%)	HPV +ve n (%)
**Pre-adolescent (9–12 yr)**	206 (23.7)	16 (7.8)	102 (18.4)	6 (5.9)	178 (29.1)	10 (5.6)	486 (23.9)	32 (6.6)
**Adolescent (13–17 yr)**	373 (43.0)	46 (12.3)	228 (41.1)	25 (11.0)	259 (42.4)	27 (10.4)	860 (42.3)	98 (11.4)
**Young adult (18–25 yr)**	289 (33.3)	52 (18.0)	225 (40.5)	43 (19.1)	174 (28.5)	37 (21.3)	688 (33.8)	132 (19.2)
**Total**	**868 (42.7)**	**114 (13.2)**	**555 (27.3)**	**74 (13.4)**	**611 (30.0)**	**74 (12.2)**	**2034 (100.0)**	**262 (12.9)**

### Prevalence of HPV infection

HPV detection was done using noninvasive urine sampling and to validate the specificity and sensitivity of the urine test for HPV, we have compared paired urine and cervical scrapes in subset of cases. Along with this we have also calculated positive and negative predictive value. The sensitivity and specificity was found to be 91.01% (95% CI = 83.05% to 96.03%) and 95.15% (95% CI = 89.03% to 98.39%) respectively while the PPV was 94.19% (95% CI = 86.94% to 98.06%) and NPV was 92.45% (95% CI = 85.66% to 96.68%) respectively. A very high concordance for HPV DNA detection was observed in both samples (k = 0.86; 95% CI: 0.79–0.94).

The prevalence of HPV infection in the three sampling states was found to be almost similar (12.9), despite distinct geographical locations and ethnicity (Table 1). Of 2034 adequate samples, 262 (12.9%) tested positive for one or more HPV genotypes. Out of 262 HPV positive girls 168 (64.1%) were found infected with HR genotypes, and almost half (132; 50.4%) of them were infected by HPV 16 alone (shown in Fig 2). There was an overall increase in HPV prevalence with age; thus, young adult girls (18–25 yrs) were having the highest (19.2%) prevalence of HPV infection followed by adolescent (11.4%) and pre-adolescent girls (6.6%) (Table 1). Interestingly, the frequency of HPV infection in tribal pre-adolescent girls was found as twice higher than what (3.3%) was reported for their urban counterparts from Delhi [12]. With regard to education, significantly a higher prevalence (15.6%) of HPV infection was observed in illiterate girls while it was lower (10.8%) in literate girls (see Table 2). The infection of HPV was higher in girls who had achieved menarche (15.3%) and accepted to having had boyfriends (14.7%) when compared to those who had neither attained menarche (6.7%) nor had boyfriends (6.7%) (Table 2). When comparison was made on economic status, a higher HPV positivity (14.6%) was observed in girls from higher income group family (Table 2). In order to reveal the level of awareness about cervical cancer and HPV, a structured questionnaire was served to all participants including their parents/guardians and teachers of the schools. The questions asked were whether they have heard about genital cancer or a virus called human papillomavirus or HPV that causes genital cancer or there is any vaccine available for prevention of this infection or not. Surprisingly, none of the participants or their parents including their teachers were aware of cervical cancer or HPV. They have never heard this before.

**Table 2:** HPV prevalence and Odds Ratio for HPV prevalence among tribal girls using multivariate analysis.

	Overall Girls (9–25 years) n = 2034 (100%)				Pre-adolescent (9–12 years) n = 486 (23.9%)				Adolescent (13–17 years) n = 860 (42.3%)				Young adult (18–25 years) n = 688 (33.8%)			
*HPV positivity*	*262 (12*.*9%)*				*32 (6*.*6%)*				*98 (11*.*4%)*				*132 (19*.*2%)*			
	N	HPV +ve n (%)	OR 95% CI	P value	N	HPV +ve n (%)	OR 95% CI	P value	N	HPV +ve n (%)	OR 95% CI	P value	N	HPV +ve n (%)	OR 95% CI	P value
**Characteristics**																
**Menarche**																
Not attained	481	32	1		274	22	1		207	10	1		0	0	1	
%	23.6	(6.7)			56.4	(8.0)			24.1	(4.8)			0.0			
Attained	1553	238	1.49 (0.96–2.27)	0.07	212	10	0.49 (0.23–1.08)	0.08	653	88	2.99 (1.51–5.9)	0.002	688	132	…….	…….
%	76.4	(15.3)			43.6	(4.7)			75.9	(13.5)			100	(19.2)		
**Boy friend**																
No	466	31	1		307	25	1		107	4	1		52	2	1	
%	22.9	(6.7)			63.2	(8.1)			12.4	(3.7)			7.6	(3.8)		
Yes	1568	231	1.52 (1.0–2.32)	0.052	179	7	0.43 (0.18–1.02)	0.056	753	94	3.54 (1.25–10.05)	0.018	636	130	5.35 (1.27–22.59)	0.022
%	77.1	(14.7)			36.8	(3.9)			87.6	(12.5)			92.4	(20.4)		
**Income**																
Low◊	1036	116	1		410	29	1		430	40	1		196	47	1	
%	50.9	(11.2)			84.4	(7.1)			50.0	(9.3)			28.5	(24)		
High◊◊	998	146	0.97 (0.74–1.29)	0.844	76	3	0.47 (0.14–1.60)	0.225	430	58	1.52 (0.99–2.33)	0.054	492	85	0.68 (0.45–1.02)	0.064
%	49.1	(14.6)			15.6	(3.9)			50.0	(13.5)			71.5	(17.3)		
**Education**																
Illiterate	891	139	1		363	27	1		318	56	1		210	56	1	
%	43.8	(15.6)			74.7	(7.4)			37.0	(17.6)			30.5	(26.7)		
Literate	1143	123	0.51 (0.39–0.67)	0.0001	123	5	0.515 (0.19–1.38)	0.187	542	42	0.372 (0.24–0.57)	0.001	478	76	0.570 (0.38–0.85)	0.006
%	56.2	(10.8)			25.3	(4.1)			63.0	(21)			69.5	(15.9)		

◊ less than 1000 INR = $20.00.

◊◊ between 2000–3000 INR = $60.00.

### HPV genotype distribution

Type-specific PCR and RLB assay analysis of HPV positive cases (262) revealed that 172 (64.1%) cases were infected with high risk types and 4 (1.5%) with probable high risk HPV types. Among them HPV 16 was found to be the most predominant type with 132 (50.4%) cases followed by HPV18 (16/232, 6.1%), HPV45 (8/262, 3.1%), HPV51 (7/262, 2.7%), HPV31 (5/262, 1.9%) and probable HR HPV type 66 (1.5%, 4/262) (see Fig 2). Interestingly, HPV16 was detected more in young adult girls (32.1%, 84/262) as compared to adolescents (17.6%, 46/262) and pre-adolescent (0.8%, 2/262) tribal girls (Table 3). Occurrence of higher prevalence of HR-HPV indicates that a large number of tribal girls are at risk of developing high grade lesions which may progress to invasive cervical cancer. Overall 34.4% (90/262) tribal girls were detected with low risk HPV types 6 and 11. Percentage of low risk types were higher in adolescent (14.1%, 37/262) and pre-adolescent (11.1%, 29/262) girls as compared to that in young adult (9.2%, 24/262) girls (see Table 3). The HPV genotyping results by RLB assay were further confirmed by DNA sequencing which very well corroborated with the type-specific PCR and RLB assay (see Table 3).

**Table 3:** Prevalence of HR- and LR-HPV genotypes in pre-adolescent, adolescent and young adult tribal girls as detected by PCR and RLB.

Categories	Overall		HR HPV types												LR HPV types			
	Number of cases	HPV +ve n (%)	HPV 16	%	HPV18	%	HPV45	%	HPV31	%	HPV 51	%	HPV 66	%	HPV6	%	HPV11	%
**Pre-adolescent (9–12 yr)**	486	32 (6.6)	2	0.8	0	0.0	0	0.0	0	0.0	0	0.0	1	0.4	16	6.1	13	5.0
**Adolescent (13–17 yr)**	860	98 (11.4)	46	17.6	5	1.9	2	0.8	3	1.1	4	1.5	1	0.4	24	9.2	13	5.0
**Young adult (18–25 yr)**	688	132 (19.2)	84	32.1	11	4.2	6	2.3	2	0.8	3	1.1	2	0.8	18	6.9	6	2.3
**Total**	**2034**	**262 (12.9)**	**132**	**50.4**	**16**	**6.1**	**8**	**3.1**	**5**	**1.9**	**7**	**2.7**	**4**	**1.5**	**58**	**22.1**	**32**	**12.2**

### Factors associated with HPV infection

Univariate analysis of 2034 tribal girls revealed that various factors studied were significantly (P<0.05) associated with HPV infection and were included in age adjusted multivariate analysis. Among three categories of girls studied, the young adults were more closely associated (Odds ratio 3.36, 95% CI, 2.25–5.05; P< 0.0001) with HPV infection as compared to adolescent (Odds ratio 1.82, 95% CI, 1.20–2.77; P< 0.005) or pre-adolescent girls (Table 4). Age adjusted analysis showed that HPV infection was strongly associated with adolescent or young adult girls (Odds ratio 2.99, 95% CI 1.51–5.9; P<0.02) who have attained menarche. Furthermore, adolescent and young adult girls having boy friends were more associated with HPV infection (Odds ratio 3.54, 95% CI 1.25–10.05; P<0.01 and Odds ratio 5.35, 95% CI 1.27–22.59; P<0.02 respectively) (Table 2). Multivariate analysis was applied on four risk factors (menarche, boyfriend, income and education) studied in order to examine which of these factors have strong association with HPV infection. It was clear from multivariate analysis that there was a strong association between HPV infection and menarche of girls (odds ratio 3.1, 95%CI, 2.03–4.73; P<0.001) who have boyfriend (Odds ratio 3.4, 95%CI 2.11–4.85; P<0.0001) (shown in Table 5). However, income and education showed weak association with HPV infection.

**Table 4:** Prevalence of HPV and their genotypes in pre-adolescent, adolescent and young adult tribal girls of India.

Categories	Number of cases	HPV +ve n (%)	OR (95% CI) P value	HR HPV types	%	LR HPV types	%
**Pre-adolescent (9–12 yr)**	486	32 (6.6)	1	3	1.1	29	11.1
**Adolescent (13–17 yr)**	860	98 (11.4)	1.825 (1.2–2.77) 0.005	61	23.3	37	14.1
**Young adult (18–25 yr)**	688	132 (19.2)	3.368 (2.25–5.05) 0.0001	108	41.2	24	9.2
**Total**	**2034**	**262 (12.9)**		**172**	65.6	**90**	34.4

**Table 5:** Multiple regression analysis of various factors studied with HPV infection in tribal girls (9–25 years).

Factors	Adjusted Odds ratio	95% CI	P value
**Menarche**	3.1	2.04–4.73	0.0001
**Boy friend**	3.41	2.11–4.85	0.0001
**Income**	1.363	1.01–1.82	0.532
**Education**	0.306	0.22–0.41	0.0001

## Discussion

Although prevalence of HPV infection is one of the world’s highest in Indian women with cervical cancer and a very low frequency of HPV infection is reported in healthy adolescent girls (3.3%) and in sexually active adult women (6–10%) [21, 28, 29], no study has been done in Indian tribal population. This is mainly due to their incommunicado habitat and lifestyle. This is a first study on Indian tribal populations showing overall significantly a higher prevalence (12.9%) of HPV infection among tribal girls aged between 9 to 25 years as compared to those reported from urban areas [21, 25, 29]. It is most interesting to note that those positive for HPV, more than 65% of them (172/262) were infected with high risk HPVs, HPV type 16 being the highest (50.4%). This trend was found to be similar in all the three states despite having different ethnic tribes, with varied social-sexual behavior and distant location.

The prevalence of HPV infection was found to be increased as a function of age; it was 6.6% in pre-adolescent girls, 11.4% in adolescents and 19.2% in young adults (Tables 1 and 2). The HPV prevalence in pre-adolescent and adolescent girls was significantly higher in comparison to those reported for urban adolescents using the same urine sampling methods. As low as 0.85% HPV infection was reported in 8–13 year pre-adolescent girls while it was only 2.3% in 13–17 year adolescent girls [21]. Another study reported only 6% infection in young adult girls aged 17–25 years from New Delhi [25] while a mean of 9.2% HPV infection was recorded in college going young girls from Tamil Nadu [29]. The frequency of HPV infection in tribal girls was significantly higher than the girls of same age group from urban area. Almost similar to our study, O’ Leary et al (2011) [30] reported 1.1% HPV prevalence in 11–14 year old urban girls however, a higher frequency of 15.2% for 15–18 year girls. Another study from Italy showed only 0.8% HPV infection in 11–14 year girls and 4.2% in 15–18 year girls but surprisingly they recorded only HR HPV type 16 [31]. A recent study conducted on Tanzanian adolescent girls of age 15–16 years who have not passed sexual debut recorded a prevalence of 8.4% of which 5.1% girls had HR HPV infection [32]. Our results also corroborate with those reported from Amerindian tribes of Brazil that demonstrated 14.3% HPV infection in sexually active tribal women aged between 10–73 years [33]. In the present study the young adult girls showed significantly a higher rate (19.2%) of HPV infection. The reasons for higher prevalence of HPV infection in Indian tribal girls may be due to their different lifestyle and sexual behavior. Occurrence of early menarche (<12 years) with high sexual permissiveness and multiple sexual partners contribute to multiple pregnancies, higher parity, malnutrition, and immunodeficiency [23] leading to increased HPV infection. It has been observed that, the risk of HPV infection was increased in adolescent girls only after sexual exposure. It is possibly because of immature genital organs of adolescents are more susceptible for contracting infections [34]. This may also partly be due to inadequate secretion of cervical mucus, which generally serves as a protective barrier against infectious agents.

It was clear from the questionnaire that almost 80% of girls who have had their partners or boyfriends showed higher prevalence of HPV infection (Table 2). There could be several other reasons for transmission of HPV from mother to child [35–37], non-penetrative sexual practices and HPV transmission via fomites [38]. HPV has also been detected in fingernail and fingertip specimens of young sexually active women [39], which might have transmitted during washing of genital area. From multivariate analysis the most significant factor appeared to be menarche, since the majority of those attained menarche and sexually active were invariably positive for HR-HPV infection.

Overall observation of significantly a higher prevalence of HPV infection in tribal girls can be attributed to their sexual behavior, lifestyle, hygiene and nutrition. They generally involve in active sexual activity at an early age (<12 years), and have multiple sexual partners leading to multiple pregnancies and higher parity. Furthermore, hygiene and cleanliness was extremely poor as they repeatedly use old cloth pieces along with cow dung ash and other unhygienic materials in their sanitary pad during menstrual period. Health problems like anemia, tuberculosis, malaria, viral fever, malnutrition and various sexually transmitted infections were very common among them. They do not have access to primary health care facilities and are highly vulnerable to various infectious and other diseases. This is compounded by poverty, illiteracy, ignorance, hostile environment, poor sanitation, lack of safe drinking water, blind beliefs and prejudices. There has been complete lack of awareness about genital cancer or HPV infections not only in participants but also among their parents and teachers.

Use of noninvasive self-collected urine sampling for HPV screening was easily acceptable to tribal girls. The exfoliated cells containing HPV DNA or HPV virions are continuously being shed from cervical epithelial linings into the urine and the DNA extracted from these cells are subjected to HPV detection by PCR. Since several soluble and insoluble renal excretions including microorganisms and enzymes present in the urine may pose problems in DNA isolation and for subsequent PCR, the methods have been modified to extract good amount of clean DNA to detect HPV by PCR [4, 21, 40, 41].

In conclusion, we demonstrate for the first time a significantly higher prevalence of HPV 16 and other HR-HPV infection in adolescent and young adult tribal girls due to different socio-sexual behaviors, early sexual debut and multiple sexual partners. Findings are indicative of a serious health concern for tribal population who may not be targeted for HPV immunization as they acquire HR-HPV infection at an early childhood but an alternative intervention may be required for better management of their reproductive health and protection from genital cancer.

## References

[1] Munoz N, Bosch FX, de Sanjose S, Herrero R, Castellsague X, Shah KV, et al Epidemiologic classification of human papillomavirus types associated with cervical cancer. N Engl J Med. 2003; 348: 518–527. 12571259 10.1056/NEJMoa021641

[2] Franceschi S, Rajkumar T, Vaccarella S, Gajalakshmi V, Sharmila A, Snijders PJ, et al Human papillomavirus and risk factors for cervical cancer in Chennai, India: A case-control study. Int J Cancer. 2003; 107: 127–133. 12925967 10.1002/ijc.11350

[3] Bharti AC, Shukla S, Mahata S, Hedau S, Das BC. Human papillomavirus and cervical cancer control in India. Expert Rev Obstet Gynecol. 2010; 5(3):329–346.

[4] Das BC, Gopalkrishna V, Sharma JK, Roy M, Luthra UK. Human papillomavirus DNA in urine of women with preneoplastic and neoplastic cervical lesions. Lancet. 1999; 340:1417–1418. 1360129

[5] Kailash U, Hedau S, Gopalkrishna V, Katiyar S, Das BC. A simple paper smear method for easy collection, transport and storage of cervical specimens for PCR detection of HPV infection. J Med Microbiol. 2002; 51: 606–610. 12132779 10.1099/0022-1317-51-7-606

[6] Pillai RM, Babu JM, Jissa VT, Lakshmi S, Chiplunkar SV, Patkar M, et al Region-wise distribution of high-risk human papillomavirus types in squamous cell carcinomas of the cervix in India. Int J Gynecol Cancer. 2010; 20:1046–1051. 10.1111/IGC.0b013e3181e02fe0 20683415

[7] Kerkar SC, Latta S, Salvi V, Pramanik J. Human Papillomavirus infection in asymptomatic population. Sex Reprod Health. 2011; 2:7–11. 10.1016/j.srhc.2010.11.001 21147453

[8] Hidesheim A, Herrero R. Human papillomavirus vaccine should be given before sexual debut for maximum benefit. J Infect Dis. 2007; 196:1431–2. 18008218 10.1086/522869

[9] U.S. Food and Drug Administration (FDA). Centre for Biologics and Evaluation research, Office of Vaccines and Related Product Applications. 2006; Available: http://www.fda.gov/BiologicsBloodVaccines/Vaccines/Approved Products/ ucm 111283

[10] Widdice LE, Brown DR, Bernstein DI, Ding L, Patel D, Shew M, et al Prevalence of human papillomavirus infection in young women receiving the first quadrivalent vaccine dose. Arch PediatrAdolesc Med. 2012; 166(8):774–6. 10.1001/archpediatrics.2012.586 22869412 PMC4042006

[11] Shew ML, Weaver B, Tu W, Tong Y, Fortenberry JD, Brown DR. High frequency of Human Papillomavirus detection in the vagina before first vaginal intercourse among females enrolled in a longitudinal cohort study. J Infect Dis. 2013; 207:1012–1015. 10.1093/infdis/jis775 23242538 PMC3571440

[12] Winer R, Lee S, Hughes J. Genital human papillomavirus infection: incidence and a risk factors in a cohort of female university students. Am J Epidemiol. 2003; 157:218–26. 12543621 10.1093/aje/kwf180

[13] Gutman LT, Herman-Giddens ME, Phelps WC. Transmission of human genital papillomavirus disease: comparison of data from adults and children. Pediatrics. 1993; 91:31–8. 8416503

[14] Mollers M, Scherpenisse M, van der Klis FR, King AJ, van Rossum TG, van Logchem EM, et al Prevalence of genital HPV infections and HPV serology in adolescent girls, prior to vaccination. Cancer Epidemiol. 2012; 36:519–524. 10.1016/j.canep.2012.07.006 22906483

[15] Dunne EF, Unger ER, Sternberg M, McQuillan G, Swan DC, Patel S, et al Prevalence of HPV Infection Among Females in the United States. JAMA. 2007; 297(8):813–819. 17327523 10.1001/jama.297.8.813

[16] Manhart LE, Holmes KK, Koutsky LA, Wood TR, Kenney DL, Feng Q, et al Human papillomavirus infection among sexually active young women in the United States: implications for developing a vaccination strategy. Sex Transm Dis. 2006; 33:502–508. 16572039 10.1097/01.olq.0000204545.89516.0a

[17] Castellsague X, Bosch FX, Munoz N, Meijer CJ, Shah KV, Sanjose DS, et al Male circumcision, penile human papillomavirus infection, and cervical cancer in female partners. N Engl J Med. 2002; 346:1105–1112. 11948269 10.1056/NEJMoa011688

[18] Das BC, Hussain S, Nasare V, Bharadwaj M. Prospects and prejudices of human papillomavirus vaccines in India. Vaccines. 2008; 26:2669–2679. 10.1016/j.vaccine.2008.03.056 18455843

[19] Gupta A, Arora R, Gupta S, Prusty BK, Kailash U, Batra S, et al Human papillomavirus DNA in urine samples of women with or without cervical cancer and their male partners compared with simultaneously collected cervical/penile smear or biopsy specimens. J ClinVirol. 2006; 37:190–194. 16931139 10.1016/j.jcv.2006.07.007

[20] Hauwers K, Depuydt C, Bogers JP, Stalpaert M, Vereecken A, Wyndaele JJ, et al Urine versus brushed samples in human papillomavirus screening: Study in both genders. Asian J Androl, 2007; 9:705–710. 17712490 10.1111/j.1745-7262.2007.00287.x

[21] Hussain S, Bharadwaj M, Nasare V, Kumari M, Sharma S, Hedau S, et al Human papillomavirus infection among young adolescents in India: impact of vaccination. J Med Virol. 2012; 84(2):298–305. 10.1002/jmv.22261 22170551

[22] Vorsters A, Micalessi I, Bilcke J, Ieven M, Bogers J, Damme PV. Detection of human papillomavirus DNA in urine. A review of the literature. Eur J ClinMicrobiol Infect Dis. 2012; 31(5):627–640. 10.1007/s10096-011-1358-z 21818524

[23] Naik E, Karpur A, Taylor R, Ramaswami B, Ramachandra S, Balasubramaniam B, et al Rural Indian tribal communities: an emerging high-risk group for HIV/AIDS. BMC Int Health Hum Rights. 2005; 5(1)–1 15723696 10.1186/1472-698X-5-1 PMC554109

[24] Mishra S, Swain BK, Babu BV. Sexual risk behaviour, knowledge and attitude related to HIV transmission: a study among a migrant tribal group living in the slums of Bhubaneswar City, Orissa, India. J Public Health. 2008; 16:331–337

[25] Prusty BK, Kumar A, Arora R, Batra S, Das BC. Human papillomavirus (HPV) DNA detection in self-collected urine. Int J Gynaecol Obstet. 2005; 90:223–227. 16043176 10.1016/j.ijgo.2005.06.004

[26] World Health Organization. Human papillomavirus laboratory manual, 1st ed. Geneva: WHO 2010; 35–63.

[27] Shukla S, Mahata S, Shishodia G, Pande S, Verma G, Hedau S, et al Physical state & copy number of high risk human papillomavirus type 16 DNA in progression of cervical cancer. Indian J Med Res. 2014; 139:531–543. 24927339 PMC4078491

[28] Bhatla N, Lal N, Bao YP, Ng T, Qiao YL. A meta-analysis of human papillomavirus type-distribution in women from South Asia: implications for vaccination. Vaccine. 2008; 26(23):2811–2817. 10.1016/j.vaccine.2008.03.047 18450340

[29] Thilagavathi A, Shanmughapriya S, Vinodhini K, Das BC, Natarajaseenivasan K. Prevalence of human papillomavirus (HPV) among college going girls using self collected urine samples from Tiruchirappalli,Tamilnadu. Arch Gynecol Obstet. 2012; 286(6):1483–6. 10.1007/s00404-012-2500-6 22886326

[30] O’Leary MC, Sinka K, Robertson C, Cuschieri K, Lyman R, Lacey M, et al HPV type-specific prevalence using a urine assay in unvaccinated male and female 11- to 18-year olds in Scotland. Br J Cancer. 2011; 104: 1221–1226. 10.1038/bjc.2011.30 21343934 PMC3068489

[31] Bianchi S, Frati ER, Panatto D, Martinelli M, Amicizia D, Zotti CM, et al Detection and genotyping of Human Papillomavirus in Urine samples from Unvaccinated Male and Female Adolescents in Italy. PLos One. 2013; 8(11):e79719 10.1371/journal.pone.0079719 24255711 PMC3821858

[32] Houlihan CF, de Sanjose S, Baisley K, Changalucha J, Ross DA, Kapiga S, et al Prevalence of Human Papillomavirus in Adolescent Girls Before Reported Sexual Debut. J Infect Dis. 2014; 210(6):837–45. 10.1093/infdis/jiu202 24740630 PMC4136803

[33] Brito EB, Martins SJ, Menezes RC. Human papillomaviruses in Amerindian women from Brazilian Amazonia. Epidemiol Infect. 2002; 128: 485–489. 12113494 10.1017/s0950268802006908 PMC2869846

[34] Moscicki AB, Hills N, Shiboski S, Powell K, Jay N, Hanson E, et al Risks for incident human papillomavirus infection and low-grade squamous intraepithelial lesion development in young females. JAMA. 2001; 285(23):2995–3002. 11410098 10.1001/jama.285.23.2995

[35] Gutman LT, Giddens ME, Phelps WC. Transmission of human genital papillomavirus disease: comparison of data from adults and children. Pediatrics. 1993; 91:31–8. 8416503

[36] Rombaldi RL, Serafini EP, Mandelli J, Zimmermann E, Losquiavo KP. Perinatal transmission of human papilomavirus DNA. Virol J. 2009; 6:83 10.1186/1743-422X-6-83 19545396 PMC2717078

[37] Lee SM, Park JS, Norwitz ER, Koo JN, Oh IH, Park JW, et al Risk of Vertical Transmission of Human Papillomavirus throughout Pregnancy: A Prospective Study. Plos One. 2013; 8(6):e66368 10.1371/journal.pone.0066368 23785495 PMC3681772

[38] Jayasinghe Y, Garland SM. Genital warts in children: what do they mean? Arch Dis Child. 2006; 91:696–700. 16670117 10.1136/adc.2005.092080 PMC2083049

[39] Winer RL, Hughes JP, Feng Q, Xi LF, Cherne S, O’Reilly S, et al Detection of genital HPV types in fingertip samples from newly sexually active female university students. Cancer Epidemiol Biomarkers Prev. 2010; 19: 1682–1685. 10.1158/1055-9965.EPI-10-0226 20570905 PMC2901391

[40] Song ES, Lee HJ, Hwang TS. Clinical efficacy of human papillomavirus DNA detection in urine from patients with various cervical lesions. J Korean Med Sci. 2007; 22:99–104. 17297259 10.3346/jkms.2007.22.1.99 PMC2693577

[41] Shigehara K, Sasagawa T, Kawaguchi S, Kobori Y, Nakashima T, Shimamura M, et al Prevalence of human papillomavirus infection in the urinary tract of men with urethritis. Int J Urol. 2010; 17:563–568. 10.1111/j.1442-2042.2010.02521.x 20345431

